# The Application of Timing in Therapy of Children and Adults with Language Disorders

**DOI:** 10.3389/fpsyg.2015.01714

**Published:** 2015-11-12

**Authors:** Elzbieta Szelag, Anna Dacewicz, Aneta Szymaszek, Tomasz Wolak, Andrzej Senderski, Izabela Domitrz, Anna Oron

**Affiliations:** ^1^Laboratory of Neuropsychology, Nencki Institute of Experimental BiologyWarsaw, Poland; ^2^University of Social Sciences and HumanitiesWarsaw, Poland; ^3^Institute of Physiology and Pathology of HearingKajetany, Poland; ^4^Children’s Memorial Health InstituteWarsaw, Poland; ^5^Department of Neurology, Warsaw Medical UniversityWarsaw, Poland

**Keywords:** temporal information processing, language, cognitive functions, aphasia, specific language disorder

## Abstract

A number of evidence revealed a link between temporal information processing (TIP) and language. Both literature data and results of our studies indicated an overlapping of deficient TIP and disordered language, pointing to the existence of an association between these two functions. On this background the new approach is to apply such knowledge in therapy of patients suffering from language disorders. In two studies we asked the following questions: (1) can the temporal training reduce language deficits in aphasic patients (Study 1) or in children with specific language impairment (SLI, Study 2)? (2) can such training ameliorate also the other cognitive functions? Each of these studies employed *pre-training* assessment, training application, *post-training* and *follow-up* assessment. In Study 1 we tested 28 patients suffering from post-stroke aphasia. They were assigned either to the temporal training (Group A, *n* = 15) in milliseconds range, or to the non-temporal training (Group B, *n* = 13). Following the training we found only in Group A improved TIP, accompanied by a transfer of improvement to language and working memory functions. In Study 2 we tested 32 children aged from 5 to 8 years, affected by SLI who were classified into the temporal training (Group A, *n* = 17) or non-temporal training (Group B, *n* = 15). Group A underwent the multileveled audio-visual computer training *Dr. Neuronowski*^®^, recently developed in our laboratory. Group B performed the computer speech therapy exercises extended by playing computer games. Similarly as in Study 1, in Group A we found significant improvements of TIP, auditory comprehension and working memory. These results indicated benefits of temporal training for amelioration of language and other cognitive functions in both aphasic patients and children with SLI. The novel powerful therapy tools provide evidence for future promising clinical applications.

## Introduction

One of the foundations in modern neuropsychology is the consistent observation that human speech has a dynamic nature and can be analyzed on different temporal levels (Pöppel, 1997). There is strong evidence supporting the thesis that temporal information processing (TIP) on both milli- and multisecond range is a critical factor for speech reception and expression, and provides an important insight into how our brains process language in norm and pathology (Szelag et al., 2004; Szelag et al., 2011a). In this report we concentrate on millisecond time range.

Experimental data showed that rapid changes in the speech signal such as formant transitions in stop-consonants (like: p, b, t, d, etc.), as well as the voice-onset-time phenomenon reflecting an asynchrony between the burst and the onset of laryngeal pulsing, are rooted in millisecond TIP. Furthermore, strong evidence indicated that various language deficits in children and adults may be associated with deficient TIP (Fink et al., 2006).

Nowadays, the notion about traditionally distinct language disorders, like aphasia, specific language impairment (SLI) or dyslexia, has been reformulated because of a close link between these syndromes. It has been believed that these impairments have similar underpinnings related to deficient TIP. In light of this evidence, the present report is focused on aphasia following cerebral infarction in adults (Study 1) and SLI in children (Study 2).

Aphasia is a consequence of brain damage and defined as an acquired complex language disorder. As only 25% of aphasic patients have a chance for full restoration of disturbed language functions, a need for new therapy methods is huge.

As indicated in pioneer reports by Efron (1963) and Swisher and Hirsh (1972), TIP deficits were evidenced in aphasic patients, independently of the modality tested (auditory or visual). The study by Wittmann et al. (2004) and Fink et al. (2006) reported that patients with deficient comprehension (Wernicke’s aphasia) displayed parallel deficits in sequencing abilities, as compared to non-fluent Broca’s patients. More recent reports confirmed deficient timing in parallel to language disability in aphasic patients (Oron et al., 2015, for a recent overview see Szeląg et al., 2015). These observations were supported by neuroanatomical data indicating an overlapping of structures controlling millisecond timing and language reception (e.g., Wittmann et al., 2004; Lewandowska et al., 2010). Despite these strong evidence, some authors doubt the association ‘*disordered timing-disordered language*’ (Bailey and Snowling, 2002; Heiervang et al., 2002; Share et al., 2002; Ramus et al., 2003). For example, Stefanatos et al. (2007) or Sidiropoulos et al. (2010) indicated either deficient or intact timing, depending on stimulus presentation procedure.

On the other hand, SLI is manifested in disturbances in normal patterns of language acquisition and delayed development of language reception or/and expression which did not result from sensory, emotional, neurological disorders or environmental factors. Although the traditional view assumed that beside language the level of other cognitive functions in SLI remains within the normal range, the more recent studies indicated important deficits in working memory, attention and executive functions (Gathercole and Baddeley, 1990; Cardy et al., 2010; Henry et al., 2012). Although the etiology of SLI remains unclear, researchers agree that deficient TIP reflecting problems in encoding both verbal and non-verbal auditory information may constitute a core deficit in SLI, at least at some cases. These observations may suggest a common neural mechanism that controls both verbal and non-verbal auditory processing which may be disordered in these children. The present paper is in line with these studies.

Starting from Tallal and Piercy (1973, 1974) who reported that children with SLI are less efficient in discrimination of both speech and non-speech sounds presented in rapid succession, recent studies confirmed difficulties in temporal order judgment for both auditory and visual tasks (Grondin et al., 2007). The other theories implicate some higher-level difficulties associated with language problems (e.g., Rice and Wexler, 1996) or procedural memory deficits (Ullman and Pierpont, 2005). The ambiguity of theories on the core deficits in SLI may be reflected in the great heterogeneity of this disorder.

To sum up, these examples confirmed the relationship ‘*timing – language’* and indicated the impaired millisecond timing as a candidate for a core symptom underlying the above two distinct language disorders, i.e., post-stroke aphasia in adults and SLI in children.

Given the neurobehavioral similarities between aphasia and SLI, some experimental studies deal with the implication of these findings in therapy programs. The idea behind such implementation is that improvement of disordered TIP through the specific exercises may result in a transfer of improvement from the trained time domain into the untrained language domain. In the existing literature there are a few training programs aiming at reducing deficits in TIP, language, attention, and short-term memory. For example, Scientific Learning developed a training program “Fast ForWord” (FFW) which is broadly spread into the clinical practice. Some studies showed that the effectiveness of computer-based interventions (e.g., FFW) in amelioration of language skills in children with SLI (Stevens et al., 2008), dyslexia (e.g., Gaab et al., 2007), and language-learning impairment (e.g., Tallal et al., 1996). The application of these trainings induces neuroplastic changes in the neural network (e.g., Hayes et al., 2003; Temple et al., 2003; Heim et al., 2013). In contrast, the other studies revealed a similar improvement after the application of FFW or other computer-based interventions in language-disordered children (e.g., Cohen et al., 2005; Gillam et al., 2008; Given et al., 2008). Inconsistency of existing results is still an open question and may be associated with individual differences in disfluency patterns, assessment of language skills, subject age or a group size in particular studies.

In our pilot study (Szelag et al., 2014) we found that in aphasic patients the application of eight sessions of temporal training during 3 weeks yielded improvement of TIP, moreover, a transfer of improvement from the trained time domain into the language domain which remained untrained during the intervention. It was evidenced in language tests assessing auditory comprehension, phoneme discrimination and voicing–unvoicing contrast detection. Importantly, the control non-temporal training did not improve either TIP or comprehension in any applied test. These data seemed promising with respect to future therapy programs addressed patients suffering from aphasia. Therefore, Study 1 presented here aimed at verification of such training effects on a larger patient sample, using longer intervention with modified parameters which may provide more massive stimulation. Furthermore, the training benefits were assessed with extended diagnostic procedures, focused not only on timing and language, but also on other cognitive functions which are also temporally segmented in the millisecond domain (Pöppel, 1994; Szelag et al., 2011a) and strongly related to the language skill (Oron et al., 2015).

The commonly occurring language disabilities in children and adults inspired us to extend our prototyping training applied in Study 1 into the complex computerized intervention program *Dr. Neuronowski*^®^. Such new therapy tool applied in Study 2 was focused not only on amelioration of TIP and language, but also other cognitive functions in which TIP is embedded. They are related to language skill and crucial for child mental activity. To test the universality of temporal training benefits we concentrated in Study 2 on SLI in children which constitutes a totally different type of language disorder than aphasia tested in Study 1, but also associated with declined TIP.

## Study 1

### Materials and Methods

#### Participants

**FIGURE 1:**
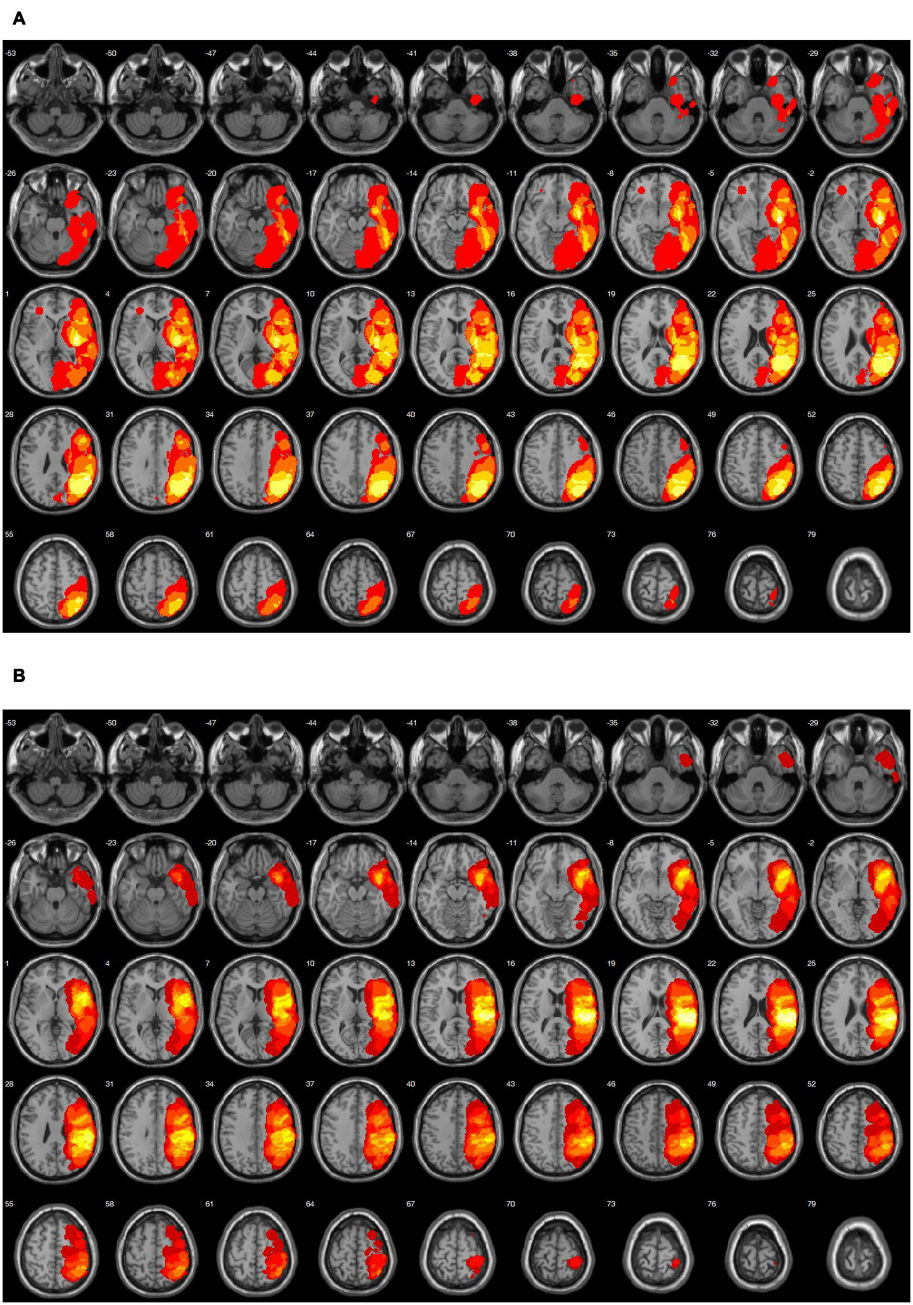
**A schematic display of the common lesioned area in Groups A and B in horizontal axis in 20 out of 28 participants. Lesions were shown with yellow/orange color. (A)** Group A (*n* = 9): the brightest color indicates the common brain damage in five subjects. It comprises left hemispheric *superior temporal gyrus, supramarginal gyrus, insula, putamen, angular gyrus.* **(B)** Group B (*n* = 11): the brightest color indicates the common brain damage in nine subjects including left hemispheric *superior temporal gyrus and supramarginal gyrus.*

Twenty eight patients suffering from aphasia after first-ever stroke (16 male, 12 female), aged between 45.8 and 78.9 years (*x* ±*SD* = 61.6 ± 9.1 years) took part in the study. All subjects were right-handed (Edinburgh Inventory), native Polish speakers and suffered predominantly from receptive language deficits, including disordered auditory comprehension following cerebral infarction (*n* = 27) or cerebral hemorrhage (*n* = 1). The location of brain damage was evidenced by CT or MRI examination (**Figure 1**). The mean lesion age was *x* ±*SD* = 4.21 ± 3.1 months. All participants had normal hearing level verified by screening pure-tone audiometry (audiometer AS 208), using frequencies ranging from 250 to 3000 Hz which covered the frequency spectrum of auditory stimuli presented in Study 1. Apart from stroke they had neither neurological nor psychiatric disorders and reported no history of head injuries or severe systemic diseases. The other exclusion criteria were recurrent stroke, global aphasia with poor verbal contact, poor general health, or participation in other rehabilitation programs during our data collection.

Auditory comprehension deficits were evidenced by seven language tests. The comprehension deficits in all subjects were accompanied by disordered TIP which was indicated by increased thresholds for auditory perception of temporal order (see below for detailed description). Moreover, working memory and attentional resources were tested in all subjects.

It was a blinded randomized controlled study. The participants were randomly assigned into two groups according to age, lesion age, level of comprehension and TIP deficits. The experimental group (Group A, *n* = 15) performed a temporal training, whereas, the control group (Group B, *n* = 13) was assigned to a non-temporal training. Synopsis of the *pre-training* performance in Group A and Group B is given in **Table 1**. Using *U* Mann–Whitney test, all between-group comparisons in the *pre-training* assessment for the above variables were non-significant with the exception of one aspect of attention which was evidenced by significantly shorter reaction times (RTs) for alertness (*p* < 0.04), corresponding to better performance in Group B than A (**Table 1**).

**Table 1:** Characteristics of two training groups in *pre-training* assessment in Study 1.

	Measurement	Group A (*n* = 15) *M (SD)*	Group B (*n* = 13) *M (SD)*	Significance (*U* Mann–Whitney test)
**Subjects data**				
	Age (*years*)	61.5 (8.3)	61.7 (10.3)	*Z* = -0.207; ns.
	Lesion age (*months*)	4.3 (3)	4.2 (3.3)	*Z* = -0.328; ns.
**Assessment**				
TIP	ATOT (*ms*)	179 (53)	166 (53)	*Z* = -1.196; ns.
Language	Token Test-36 (*% of errors*)	48.3 (22.6)	50.2 (26.4)	*Z* = -0.322; ns.
	PDPseudo (*% of errors*)	21.1 (14.9)	15.4 (9.3)	*Z* = 1.001; ns.
	PD Words (*% of errors*)	14 (13.9)	12.5 (11.3)	*Z* = -0.123; ns.
	PHNoise (*% of errors*)	19.6 (7.2)	23.4 (10.5)	*Z* = -0.874; ns.
	PHComp (*% of errors*)	15.2 (8.9)	17 (9.4)	*Z* = -0.438; ns.
	IT (*% of errors*)	55.7 (37.2)	68 (42.3)	*Z* = -1.300; ns.
	ComprVC (*% of errors*)	38 (22.1)	43.9 (38.4)	*Z* = -0.093; ns.
**Other cognitive functions**				
Working memory	SSP (*total score*)	4.2 (0.94)	4.5 (1.1)	*Z* = -0.139; ns.
	SWM (*total score*)	51.9 (26.4)	44.6 (23.1)	*Z* = -0.853; ns.
Attention	Alertness (*RT in ms*)	341 (89)	272 (43)	*Z* = -2.10; *p* = 0.04
	Vigilance (*RT in ms*)	741 (213)	655 (134)	*Z* = -0.931; ns.

*PDPseudo, Phoneme Discrimination for Pseudowords; PDWords, Phoneme Discrimination for Words; PHNoise, Phonemic Hearing in Noise; PHComp, Phonemic Hearing for Compressed Speech; IT, Inflection Trails; ComprVC, Comprehension of Verbal Commands; SSP, Spatial Span; SWM, Spatial Working Memory.*

##### Neuroanatomical verification of brain damage and between-group balance

To verify the brain lesions structural data (CT or MRI) were conducted. The neuroanatomical data confirmed that the lesioned area in Group A (**Figure 1A**) and Group B (**Figure 1B**) comprised almost the same left hemispheric structures, covering the classical areas engaged in both auditory comprehension and TIP (Wittmann et al., 2004; Szelag et al., 2009; Lewandowska et al., 2010). It may be assumed that Group A and Group B were as matched as possible (**Table 1**, **Figure 1**).

#### Ethical Approval

The study protocol was approved by the Bioethical Commission at the Medical University of Warsaw (permission no *KB/5/2010*), as well as by the Ethic Commission at the University of Social Science and Humanities (permission no *2/III/07-08*). The study was conducted according to the Helsinki Declaration; the written informed consent from each participant was obtained prior to the testing.

#### Materials and Procedures

The study comprised both assessment and training procedures. The assessment battery included evaluation of TIP, language and other cognitive functions which could influence language skills. The computerized training procedures comprised temporal training (experimental Group A) and non-temporal control training (Group B).

##### Assessment battery

*Assessment of TIP* focused on sequencing abilities in millisecond domain and based on measurement of auditory temporal-order threshold (ATOT; Szymaszek et al., 2009; Szelag and Skolimowska, 2012; Szelag et al., 2014). To summarize briefly, ATOT is defined as the minimum time gap between two auditory stimuli presented in rapid succession that is necessary for a participant to report correctly their order, i.e., the relation *before–after* at 75% correctness. The stimuli were paired 1 ms clicks presented monaurally, i.e., one to each ear with various Inter-Stimulus-Intervals (ISIs). The task was to report the order of these two clicks by pointing to one of two response cards indicating the presentation order: *left–right* or *right–left*. ISI varied adaptively from 1 to 600 ms according to the adaptive maximum-likehood-based algorithm YAAP (Treutwein, 1997). The ISIs in each trial were set at the current best estimate of the ATOT. This tracking procedure estimates a threshold corresponding to 75% correct order detection based on a logistic psychometric function. The stimulus presentation was terminated when the location of the ATOT was located with a probability of 95% inside a ± 5 ms interval around the currently estimated threshold.

The stimuli were generated by a 16-bit Sound Blaster Extigy Card and delivered through headphones at a comfortable listening level. Each paired-stimulus was preceded by a warning signal. The proper data collection followed the introductory session (described in Szelag et al., 2014). ATOTs were collected in two sessions conducted in consecutive days.

Outcome measure: the mean ATOT from two sessions (in ms).

*Assessment of language functions.* *Token Test* (Huber et al., 1983) is sensitive to deficits in auditory comprehension that are one of the main aphasic symptoms. The task was to follow spoken commands of increasing length and complexity. Patients’ responses were given either by pointing to, or manipulating with plastic tokens (colored squares and circles of two sizes: big/little), e.g., *“Touch the white circle after taking away the yellow square.”* The whole test consisted of 50 commands given in five sections of increasing complexity.

*Phoneme Discrimination for Pseudowords* (*PDPseudo*): the task was to decide whether two paired pseudowords were the same or different indicating one of two response cards. The pseudowords differed in consonants, contrasting to place of articulation, fricative, voicing, and nasality, as well as in consonant omission or shifting. The entire test comprised 35 paired pseudowords presented in seven series (75% of pairs were different/25% the same).

*Phoneme Discrimination for Words* (*PDWords*; Nowak-Czerwinska, 1994) was similar to PDPseudo (see above). The only difference was that paired pseudowords were substituted with paired words. The entire test comprised 64 pairs of words presented in eight series (75% different/25% the same).

*Phonemic Hearing in Noise* (*PHNoise*; modified version of Phonemic Hearing Test, Szelag and Szymaszek, 2006). The measurement comprised phoneme discrimination in sentences presented *via* headphones on the background of a cocktail-party noise. Following each sentence presentation, the participant was asked to point to one of two pictures corresponding to that sentence. For example, the sentence “*This snake is black*” was presented and the patient pointed to one of two pictures presenting either black snake (in Polish *: wąż*) or black mustache (in Polish: *wąs*). The test consisted of 106 trials.

*Phonemic Hearing for Compressed Speech* (*PHComp*; modified version of Phonemic Hearing Test, Szelag and Szymaszek, 2006). Instead of the background noise, we applied sentences compressed by 20% which made the task more difficult. The experimental protocol was identical as that used for PHNoise.

*Inflection Trials* (*IT;* Lucki, 1995; Szepietowska, 2000) assessed auditory comprehension of grammar structures using sentences in which one or more grammatical categories with declension (nouns) and suffixes were expressed. In inflection the same word appears in different grammatical forms changing the sentence meaning. The task was to follow spoken commands in six trials, e.g.: “*Please, point to the pencil with the key*” (In Polish: “*Proszę, pokaż ołówek kluczem”*) and the inflected one: “*Please, point to the key with the pencil*” (in Polish: “*Proszę, pokaż klucz olówkiem”*).

*Comprehension of Verbal Commands* (*ComprVC;* Lucki, 1995; Szepietowska, 2000) assessed the spatial orientation in the body schema, and understanding of the body parts (i.e., forehead, ears, eyes). The task was to perform five commands, e.g.: “*Please, point to your nose with the left index finger.*” *Outcome measures in all language tests: percentage of errors committed in particular tests.*

*Assessment of other cognitive functions. Working memory (CANTAB, Cambridge Cognition, 2005).* *Spatial Span* (*SSP*) measured working memory span in a computerized version of the Corsi Block Test. Various sequences of squares was presented on the screen. The task was to remember the square sequence and to touch the squares in the same order as presented. The number of squares presented within the sequence increased from two to nine. If the subject did not point to the correct sequence in three consecutive trials, the test was terminated. The longest sequence of squares reflected the span length.

Outcome measure: the number of memorized items.

*Spatial Working Memory* (*SWM*) required retention and manipulation of visuo-spatial information. The task was to find blue ‘tokens’ in presented boxes and use them to fill up an empty column on the right side of the screen.

*Outcome measure: the number of committed errors* (i.e., touching empty boxes and revisiting boxes which already were found to contain a token).

*Attention (Test of Attentional Performance; Zimmermann and Fimm, 1997)*. *Alertness* required simple RTs measurement in response to a visual stimulus (a white cross displayed in the screen center) which in half trials was preceded by an auditory warning signal. The task was to press the response pad after presentation of the cross.

*Vigilance.* A low (440 Hz) and a high (1000 Hz) tone were presented sequentially in a random order during 10-min session. The task was to press a response pad when two identical tones were presented in a row.

Outcome measure in two attention tests: mean RT achieved in particular test.

##### Training procedures

###### Temporal training

The temporal training used our prototyping procedure developed in previous studies (Szelag et al., 2014). It was rooted in improvement of sequential abilities in the millisecond timing. The main idea of this procedure was complementary to that applied in the assessment of ATOT (see above). Accordingly, the patient was asked to report the order of two clicks presented in rapid sequences with various ISIs. In such a task the shorter ISI corresponded to more difficult task. At the starting point the task difficulty of the training was individually adjusted for each participant on a basis of his/her *pre-training* ATOT (**Table 1**). The training was provided in 10-trial blocks with fixed ISI in each block. ISIs varied between blocks depending on the score of correct/incorrect responses according to an algorithm based on the patient’s actual ATOT. When at least 90% of correct responses in a block was achieved, the ISI in following block was decreased (increasing task difficulty) according to the following rules: (1) if the actual ATOT was longer than 100 ms, ISI decreased by 5 ms; (2) in case of ATOT between 50 and 100 ms ISI decreased by 2 ms; (3) for ATOT below 50 ms by 1 ms. In case of correct score within a block below 90%, the ISI increased (decreasing task difficulty) according to the same algorithm as described in (1), (2), and (3).

After each subject’s response a visual feedback on correctness was provided. Additionally, an extra motivation reward system was applied. The participant obtained 1 point after each correct response, 1 point was subtracted in case of incorrect response. After completion of entire 10-trial block the number of collected points was displayed on the screen. Finally, following completion of each block, the participant was rewarded with puzzles which were collected on the monitor during the training session.

###### Non-temporal control training

The control training was based on loudness discrimination without any aspect of millisecond TIP trained above. Paired 1 s tones separated by a constant ISI of 3 s were presented *via* headphones. One paired tone was always louder than the other tone. The task was to report which tone was louder: the first one or the second one. The adaptivity in the control training based on the loudness difference within paired tones. The loudness of paired tones differs within 0.025–0.00025 of the amplitude range with a constant step of 0.00025. The training was provided in 10-trial blocks. Moreover, four frequencies were used in presented tones: 400, 600, 800, and 1000 Hz, nevertheless within each block only one frequency of presented stimuli was used. In the control training the same motivation system, protocol, as well as comparable mental load was applied as in the temporal training.

##### Study protocol

**FIGURE 2:**
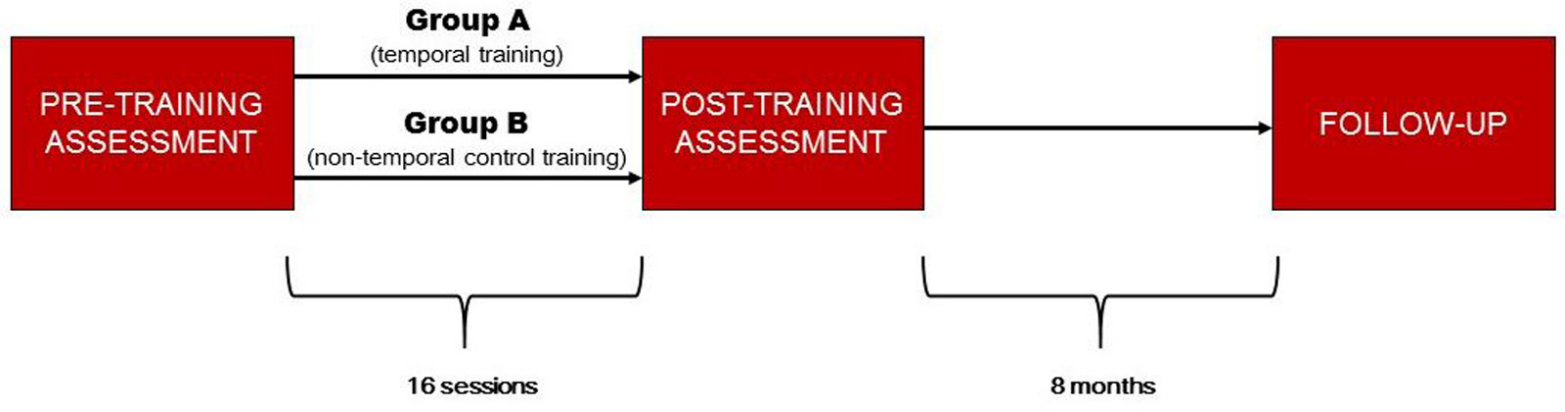
**Experimental protocol applied in Study 1**.

All assessment procedures in each patient were conducted before and after the training (**Figure 2**). Subsequently, each patient performed individually under supervision of an experimenter 16 sessions of the temporal (Group A) or control (Group B) training for 5 weeks – three sessions per week, each session lasted 45 min. The stability of improvements was verified eight months after the training completion.

##### Statistical analyses

To verify the effects of each training type on TIP, language and the other cognitive functions (*pre- vs. post-training* performance), as well as the stability of these effects *(follow-up vs. post-training)* Wilcoxon Signed-Rank test for two dependent samples (within-group comparisons) was performed.

### Results

**FIGURE 3:**
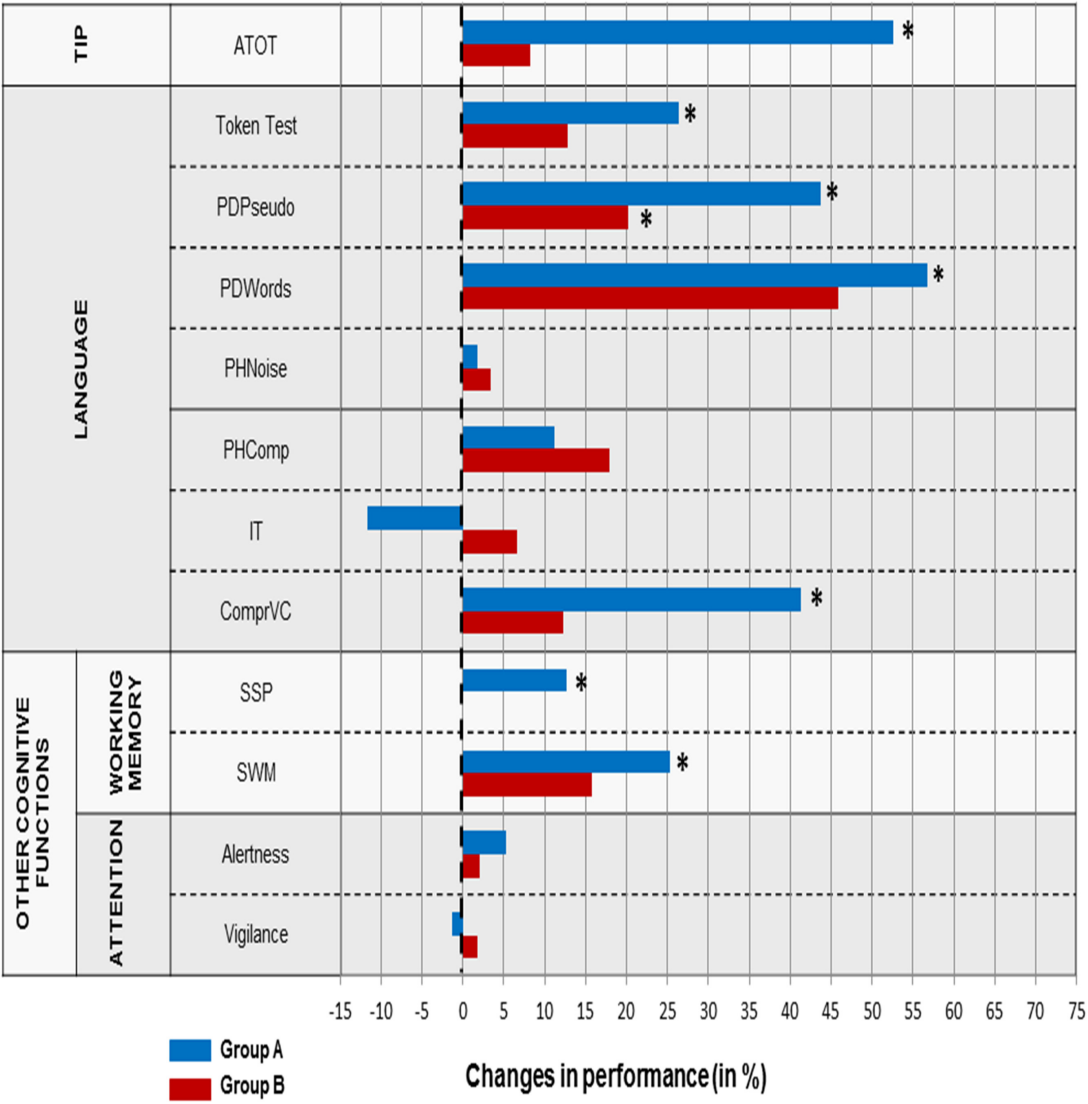
**Difference in % in the level of *pre- vs. post-training* performance in Study 1 for particular tasks in Group A and B.** The dashed vertical line reflects a stable performance (no difference between *pre- and post-training* performance). The positive values (on the right from the dashed vertical line) reflect the improved performance after the training. The minus values (on the left from the dashed line) display the worsened performance. Significant differences (*p* < 0.05) are indicated by asterisks.

The effect of temporal and non-temporal training was evaluated for particular tasks. The profile of changes in *pre- vs. post-training* performance is given on **Figure 3**.

#### Timing, language and other cognitive outcomes following two training types: *pre- vs. post-training* comparisons

##### Temporal Information Processing

*ATOT:* the threshold values *pre-training* (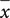 = 179 ms) were significantly higher (*Z* = -3.408, *p* < 0.001) than those *post-training* (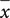 = 85 ms) in Group A. Such difference in Group B was non-significant (166 ms *vs.* 153 ms in *pre-* and *post-training*, respectively).

##### Language functions

*Token Test*: the percentage of errors *pre-training* (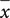 = 48%) was significantly higher (*Z* = -2.487, *p* < 0.02) than that *post-training* (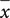 = 35.6%) in Group A. Such difference Group B was non-significant (50 *vs.* 43.8% in *pre-* and *post-training*, respectively).

*PDPseudo:* the percentage of errors *pre-training* (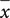 = 21%) was significantly higher (*Z* = -2.955, *p* < 0.004) than that *post-training* (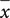 = 11.9%) in Group A. The similar relationship (*Z* = -2.355, *p* < 0.02) was found in Group B (15 *vs.* 12.6% in *pre-* and *post-training*, respectively).

*PDWords:* the percentage of errors *pre-training* (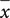 = 14%) was significantly higher (*Z* = -2.950, *p* < 0.004) than *post-training* (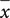 = 6%) Group A. Such difference in Group B was non-significant (12.5 *vs.* 6.8% in *pre-* and *post-training*, respectively).

*PHNoise*: the difference in the percentage of errors committed in the *pre- vs. post-training* assessment in both groups was non-significant. The mean percentage of committed errors in Group A was 19.6 *vs.* 19.2% in *pre-* and *post-training*, respectively, whereas in Group B it was 23.4 *vs.* 22.6%, respectively.

*PHComp:* the difference in the percentage of errors *pre- vs. post-training* was non-significant in both groups. The mean percent of errors in Group A was 15.3 *vs.* 13.5% for *pre-* and *post-training*, respectively and in Group B it was 17 *vs.* 13.9%, respectively.

*IT:* the difference in the percentage of errors in the *pre- vs. post-training* assessment was non-significant in both groups. The mean percentage of errors committed in Group A was 51.7 *vs.* 57.7% in *pre-* and *post-training*, respectively and in Group B it was 67.9 *vs.* 63.5%, respectively.

*ComprVC:* the percentage of errors *pre-training* (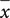 = 38%) was significantly higher (*Z* = -2.144, *p* < 0.04) than that *post-training* (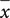 = 22.3%) in Group A. Such difference in Group B was non-significant (43.9 *vs.* 38.5% in *pre- vs. post-training*, respectively).

##### The other cognitive functions

###### Working memory

*SSP:* the number of memorized items (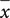 = 4.2) in *pre-training* assessment was significantly lower (*Z* = -1.999, *p* < 0.05) than that *post-training* (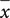 = 4.7) in Group A. Such difference in Group B was non-significant (4.5 *vs.* 4.5 for *pre-* and *post-training*, respectively).

*SWM:* the number of errors *pre-training* (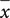 = 51.9%) was significantly higher (*Z* = -2.358, *p* < 0.02) than that *post-training* (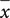 = 38.7%) in Group A. Such difference in Group B was non-significant (44.6 *vs.* 37.6% in *pre-* and *post-training*, respectively).

###### Attention

*Alertness:* the difference in the mean RT achieved in *pre-training* assessment compared to *post-training* assessment was non-significant in both groups. The mean RT in Group A was 341 *vs.* 323 ms in *pre-* and *post-training*, respectively and in Group B it was 272 *vs.* 267 ms, respectively.

*Vigilance:* the difference in the mean RT achieved *pre- vs. post-training* was non-significant in both groups. The mean RT in Group A was 741 *vs.* 751 ms for *pre-* and *post-training*, respectively and in Group B it was 655 *vs.* 643 ms, respectively.

#### Stability of Improvements Obtained in Group A

The stability of obtained improvements (*follow-up vs. post-training* performance) was assessed only in Group A for tasks in which significant improvement *post- vs. pre-training* was evidenced. The *follow-up* assessment in Group A was performed in 10 patients due to the worsened health status in the other ones.

To summarize, the lack of significant differences between *the follow-up* and *post-training* assessment confirmed relatively stable improvements in Group A. It indicated the benefits of temporal training for timing, language and cognitive functions. The obtained outcome measures indicated the continuous progress in subjects’ behavior following the temporal training (**Table 2**).

**Table 2:** Summarized results of stability of improvement in Study 1 in Group A *follow-up vs. post-training* comparisons.

	Group A						
		*n*	*Post- M (SD)*	*Follow-up M (SD)*	*Z*	*p*
TIP	ATOT	6	68 (34)	83 (26)	-1.153	ns.
Language	Token Test	10	32.2 (30)	26.4 (26)	-1.183	ns.
	PDPseudo	10	12.1 (9.9)	10.9 (8.9)	-1.245	ns.
	PDWords	10	7.5 (16.3)	7.5 (11.7)	-0.282	ns.
	ComprVC	4	22.5 (25)	20 (0)	c	
**Other cognitive functions**						
Working memory	SSP	7	4.8 (0.6)	5 (0.5)	-1.000	ns.
	SWM	9	39.1 (20.5)	41 (14.8)	-0.178	ns.

*As in the follow-up assessment some patients did not perform the full set of tasks, the number of tested participants is given at each task. c, statistical analysis was not performed because of insufficient number of subjects. Values of ATOT are provided by ms; Token Test, PDPseudo, PDWords and ComprVC by % of errors; SSP and SWM by total score.*

#### Summary of Results

The application of the temporal training in aphasic patients ameliorated significantly TIP which was reflected in lower ATOT values, language skill evidenced in Token Test, PDPseudo, PDWords, and ComprVC, as well as working memory capacity verified in SSP and SWM. Following the temporal training no significant improvement was reported in PHNoise, PHComp, IT, as well as in Alertness and Vigilance of attention. On the other hand, following the non-temporal training in Group B no significant improvement was observed in any of the applied test except PDPseudo. All reported improvements were relatively stable for 8 months after the temporal training.

## Study 2

### Materials and Methods

#### Participants

Thirty two children (10 girls, 22 boys) suffering from SLI (F.80.1 and F.80.2 according to ICD 10; World Health Organization, 1992) aged between 5 and 8 years participated in the study. They were recruited at the Early Intervention Centre and the Children’s Memorial Health Institute in Warsaw. All subjects were right-handed (Edinburgh Inventory) and native Polish speakers. The language delay was defined as a reduced performance evidenced by the Test for Assessment of Global Language Skills (TAGLS, Tarkowski, 2001) which constitutes the screening assessment for language development in Polish children. All participants obtained the overall standard score or at least two standard subtests below or equal fourth sten. All children represented the normal level of non-verbal intelligence (IQ at least 85 or higher, measured by the Raven’s Colored Progressive Matrices, Szustrowa and Jaworowska, 2003) and normal hearing level (screening pure-tone audiometry, audiometer AS 208) for frequencies ranging from 500 to 4000 Hz which covered the frequency spectrum of auditory stimuli presented in Study 2. The exclusion criteria were neurological, psychiatric, socio-emotional or attentional disorders (as determined by the parental report), as well as the participation in other therapy during our data collection.

It was a blinded randomized controlled study. The recruited children were randomly assigned into two groups (experimental and control) according to age, gender, non-verbal IQ and the level of language development. The experimental group (Group A, *n* = 17) underwent the temporal training and the control group (Group B, *n* = 15) obtained the non-temporal training. Using *U* Mann–Whitney test the two groups did not differ significantly in *pre-training* assessment for the tested variables (**Table 3**).

**Table 3:** Characteristics of two training groups in the *pre-training* assessment in Study 2.

	Measurement	Group A (*n* = 17) *M (SD)*	Group B (*n* = 15) *M (SD)*	Significance (*U* Mann–Whitney test)
**Subjects data**				
	Age (*years*)	6.2 (1)	5.9 (0.7)	*Z* = 0.370; ns.
	IQ	102.9 (30)	113.1 (16.9)	*Z* = 0.313; ns.
	Language development TAGLS (*stens*)	2.9 (1.8)	2.7 (1.3)	*Z* = 0.186; ns.
**Assessment**				
TIP	ATOT (*ms*)	196 (6)	211 (76)	*Z* = -0.672; ns.
Language	Token Test-36 (*% of errors*)	52.9 (25.2)	59.8 (21.1)	*Z* = -0.892; ns.
	PDPseudo (*% of errors*)	34.3 (13.6)	32.3 (12.4)	*Z* = -0.546; ns.
	PDWords (*% of errors*)	18.9 (13)	21 (14.1)	*Z* = -0.379; ns.
	SSC (*% of errors*)	34.6 (28.7)	33.3 (24.3)	*Z* = -0.076; ns.
	COWAT (*number of words*)	9.6 (5.6)	7.1 (3.5)	*Z* = -1.404; ns.
**Other cognitive functions**				
Working memory	SSP (*numbers of errors*)	9.4 (2.5)	12.3 (6)	*Z* = -0.022; ns.
	Digit Span (*total score*)	1.7 (0.8)	2.1 (0.6)	*Z* = -1.158; ns.
	VWM (*total score*)	6 (2)	5.3 (1.6)	*Z* = -0.835; ns.
Attention	Alertness (*RT in ms*)	438 (155)	481 (161)	*Z* = -1.322; ns.
	Mazes (*total score*)	20.4 (6.9)	17.5 (6.0)	*Z* = -1.331; ns.
Executive functions	TOL^DX^ (*total move score*)	55.6 (19.7)	64.8 (16.9)	*Z* = -1.559; ns.

*TAGLS, Test for Assessment of Global Language Skills; PDPseudo, Phoneme Discrimination for Pseudowords; PDWords, Phoneme Discrimination for Words; SSC, Syntactic Structure Comprehension; COWAT, Controlled Oral Word Assessment Test; SSP, Spatial Span; VWM, Verbal Working Memory, TOL^DX^, Tower of London Drexel University.*

#### Ethical Approval

The study protocol was approved by the Bioethical Commission at the Medical University of Warsaw (permission no. KB/162/2010). Written informed consent was obtained from the parents of each child participating in the study, children provided verbal approval.

#### Materials and Procedures

Similarly to Study 1, Study 2 comprised both assessment and training procedures. The assessment battery included TIP, language and other cognitive functions e.g., working memory, attentional resources and executive functions. The computerized training procedures comprised temporal training (Group A) and non-temporal control training (Group B).

##### Assessment battery

*Assessment of TIP* based on the same procedure as applied in Study 1, the only difference was that ATOTs were collected from one session only.

###### Assessment of language functions

*Token Test-36* (Kosciesza and Krasowicz, 1995) was a modified version of Token Test for adults used in Study 1 (the whole tests consisted of 30 commands).

*Phoneme Discrimination for Pseudowords* (*PDPseudo*): the measurement used the same procedure as applied in Study 1.

*Phoneme Discrimination for Words* (*PDWords*; modified version of Szelag and Szymaszek, 2006): the measurement used the same procedure as applied in Study 1.

*Syntactic Structures Comprehension* (*SSC*; unpublished materials elaborated in our laboratory): participants listened to 40 sentences classified into 10 series. Each series contained a set of four sentences of a similar meaning, but differing in either (1) plural *vs.* singular form or (2) a preposition of place. The task was to indicate on the response card the picture corresponding to one of these four syntactic situations (e.g., *”the elephant is standing…in/next to/in front of/ behind…the tent”)*.

*Outcome measures in all above language tests was the percentage of errors committed in particular tests*.

*Controlled Oral Word Assessment Test* (*COWAT*; Lezak, 1995) measured verbal fluency. The task was to produce as many words as possible from the category of animals during 1 min.

Outcome measure: the number of produced words.

###### Assessment of other cognitive functions

*Working memory. Spatial Span* (*SSP*; CANTAB; Cambridge Cognition, 2005) the same procedure as described in Study 1 was proceeded, nevertheless the outcome measure analyzed here was the number of committed errors.

*Digit Span* (Wechsler Intelligence Scale for Children – Revised Version; WISC-R; Matczak et al., 1991): the task was to listen to the sequence of numbers and recall them in the same order. The number of digits increased from three up to nine. For each correctly reproduced series one point was awarded.

Outcome measure: total score in the entire test.

*Verbal Working Memory Test* (*VWM*; unpublished materials elaborated in our laboratory): the task was to reproduce the order of listened unrelated words presented in series (ranging in words number from two to nine), by pointing to pictures corresponding to presented words. In the first part the words were phonologically similar, whereas, in the second one they were phonologically dissimilar. One point was awarded for each correctly reproduced series.

Outcome measure: total score in the entire test.

###### Attentional resources

*Alertness* (Zimmermann et al., 2005): the task was to press a button as fast as possible when the target (picture of a witch presented in a computer screen) appeared in a castle window.

The outcome measure: the median of RT achieved in the entire test.

###### Executive functions

*Mazes* (WISC-R; Matczak et al., 1991): the task was to solve 9 maze puzzles of increasing difficulty in a given time limit from 30 to 150 s. For each correctly solved maze, 4 points were awarded. In case of an error one point was subtracted. The error comprised choosing wrong way or passing through the wall.

Outcome measure: total score in the entire test.

*Tower of London Drexel University* (*TOL^DX^*; Culbertson and Zillmer, 2005*)* consisted of two identical tower structures, one for the subjects and the other for the examiner. Each structure consisted of a board with three pegs and a set of three beads on pegs (red, green, and blue). The task was to replicate 10 configurations of the beads presented by the examiner in as few moves as possible, following two rules: (1) prohibited placement of more beads on a peg than it was accommodated, and (2) to move the beads from pegs one at time.

*Outcome measure: the total move score, reflecting the number of additional moves made while replicating the beads configuration*.

##### Training procedures

*Temporal training* procedure used the multimedia intervention program called *Dr. Neuronowski*^®^ (www.neuronowski.com) designed in our laboratory. This software consists of nine modules containing 46 games. The majority of these games involved millisecond TIP, sequencing abilities and duration judgment based on results of our previous studies. They were extended by training of other cognitive functions. The task difficulty in particular games changed adaptively on the basis of the actual level of child’s performance. The task difficulty comprised: number, length or rate of presented stimuli, various ISIs, the rate of modified speech, application of distractors and time limits for child’s responses. The software was designed for tablets, to make it more attractive for children. Particular modules aimed to train the following functions: attention and non-verbal auditory perceptual abilities (*Module 1)*, millisecond TIP (*Module 2*, tasks were complementary to both the assessment of ATOT and our prototyping training applied in Study 1), working memory (*Module 3)*, executive functions *(Module 4* and *8)*, receptive language and phonemic hearing (*Module 5* and *6)*, duration judgment of short sounds (*Module 7)*, phonemic hearing (*Module 9*) using the Voice-Onset-Time phenomenon (Szelag and Szymaszek, 2014).

###### Non-temporal training

Control training comprised three computer speech-therapy games and 16 computer games available in the Internet (e.g., *Memory or Tetris*), performed on tablets. Speech-therapy games trained phonemic hearing, articulation and vocabulary. The computer games trained attention, working memory and executive functions. Contrary to the temporal training, these tasks did not involve any exercises in rapid auditory processing.

##### Study protocol

All assessment procedures were conducted before and after the intervention (**Figure 2**). Each child performed individually, under supervision of an examiner, 24 training sessions of the temporal (Group A) or non-temporal training (Group B) for 6 weeks, four sessions per week, each session lasted 60 min. The stability of improvement was verified in the *follow-up* assessment 6 weeks after the training completion.

### Results

**FIGURE 4:**
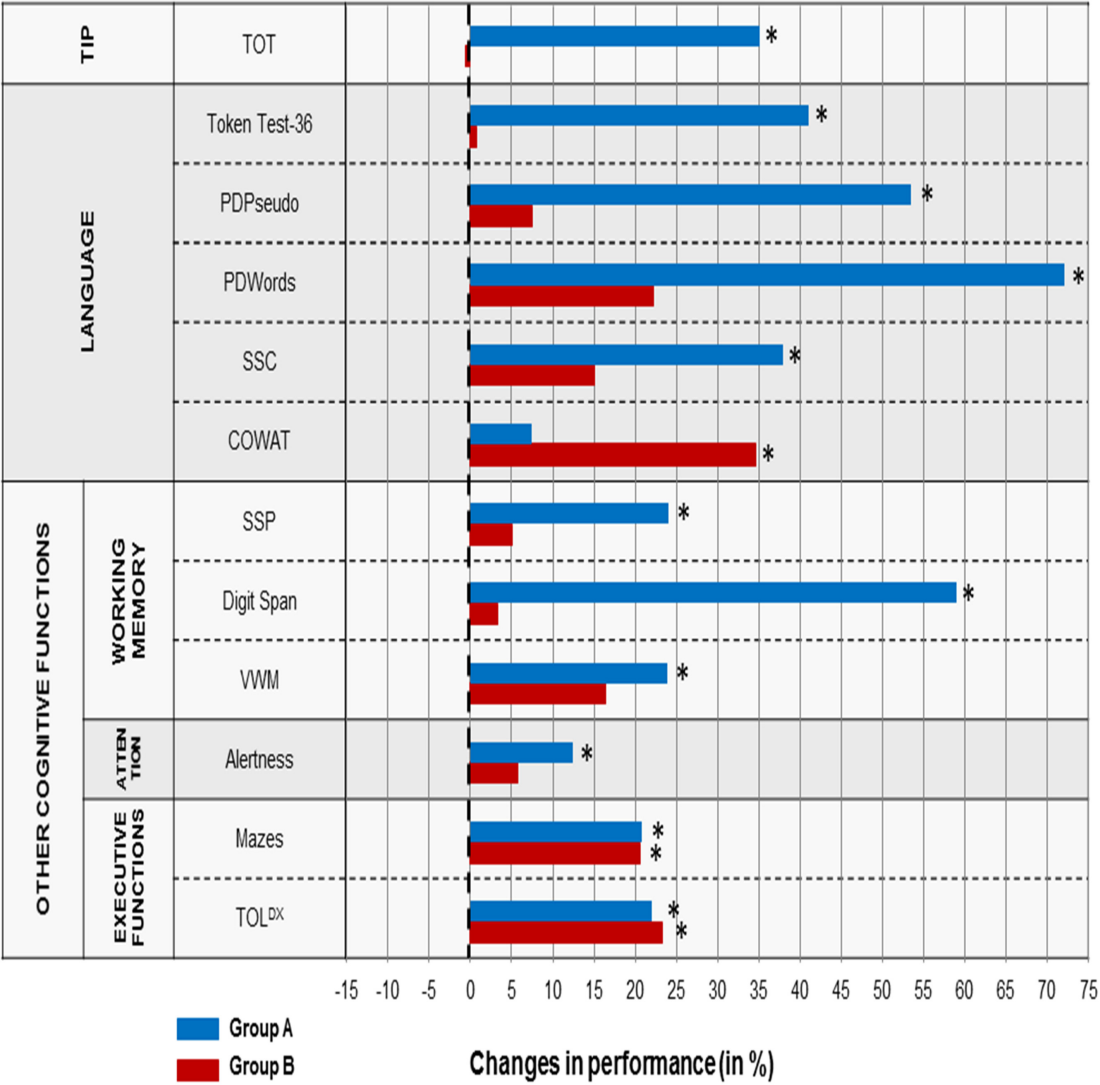
**Difference in % in the level of *pre- vs. post-training* performance in Study 2 for particular tasks in Group A and B.** The dashed vertical line reflects a stable performance (no difference between *pre-* and *post-training* performance). The positive values (on the right from the dashed vertical line) reflect the improved performance after the training. The negative values (on the left from the dashed line) display the worsened performance. Significant differences (*p* < 0.05) are indicated by asterisks.

Similarly to Study 1, the effects of each training type on TIP, language and other cognitive functions (*pre- vs. post-training* performance), as well as the stability of these effects *(follow-up vs. post-training)* was assessed using Wilcoxon Signed-Rank test for two dependent samples (within-group comparisons). The profile of changes in *pre- vs. post-training* performance for each task is given on **Figure 4**.

#### Timing, Language and Other Cognitive Outcomes Following Two Training Types: *Pre- vs. Post-training* Comparisons

##### Temporal Information Processing

*ATOT* values in Group A were significantly higher (*Z* = -3.464; *p* < 0.001) *pre-training* (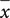 = 196 ms) than *post-training* (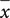 = 127 ms). Such difference in Group B was non-significant (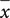 = 211 ms *vs.* 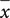 = 225 ms for *pre-* and *post-training*, respectively).

##### Language functions

*Token Test-36:* the percentage of errors in Group A was significantly higher (*Z* = -3.524; *p* < 0.001) *pre-training* (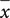 = 52.9%) than *post-training* (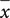 = 31.2%). Such difference in Group B was non-significant (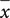 = 59.8% *vs.* 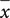 = 55.6% for *pre-* and *post-training*, respectively).

*PDPseudo:* the percentage of errors in Group A was significantly higher (Z = -2.708; *p* < 0.007) *pre-training* (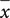 = 34.3%) than *post-training* (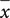 = 16%). Such difference in Group B was non-significant (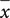 = 32.3% *vs.* 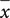 = 29.9% for *pre-* and *post-training*, respectively).

*PDWords:* the percentage of errors in Group A was significantly higher (*Z* = -3.626; *p* < 0.001) *pre-training* (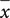 = 18.9%) than *post-training* (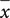 = 5.3%). Such difference in Group B was non-significant (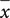 = 21% *vs.* 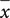 = 16.3% for *pre-* and *post-training*, respectively).

*SCC:* the percentage of errors in Group A was significantly higher (*Z* = -2.877; *p* < 0.004) *pre-training* (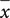 = 34.6%) than p*ost-training* (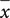 = 21.5%). Such difference in Group B was non-significant (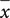 = 33.3% *vs.* 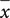 = 28.3% for *pre-* and *post-training*, respectively).

*COWAT:* the difference between the number of produced words in Group A was non-significant between *pre-training* (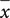 = 9.6) and *post-training* (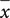 = 10.4). In Group B the number of produced words was significantly lower (*Z* = -0.825; *p* < 0.02) *pre-training* (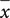 = 7.1) than *post-training* (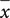 = 9.6).

##### The other cognitive functions

###### Working memory

*SSP:* the number of errors in Group A was significantly higher (*Z* = -2.753; *p* < 0.05) *pre-training* (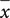 = 12.4) than *post-training* (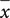 = 9.4). Such difference in Group B was non-significant (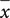 = 12.2 *vs.* 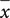 = 12.8 for *pre-* and *post-training*, respectively).

*Digit Span:* the total score in Group A was significantly lower (Z = -2.640; *p* < 0.008) *pre-training* (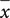 = 1.7) than *post-training* (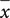 = 2.7). Such difference in Group B was non-significant (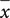 = 2.1 *vs.* 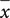 = 2.1 for *pre-* and *post-training*, respectively).

*VWM:* the total score in Group A was significantly lower (Z = -2.620; *p* < 0.009) *pre-training* (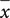 = 6) than post*-training* (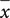 = 7.4). Such difference in Group B tended toward significance (*Z* = -1.922; *p* < 0.055; 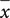 = 5.3 *vs.* 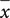 = 6.1 in *pre-* and *post-training*, respectively).

##### Attention

*Alertness:* median RT in Group A was significantly longer (Z = -2.676; *p* < 0.007) *pre-training* (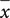 = 438 ms) than *post-training* (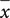 = 384 ms). Such difference in Group B was non-significant (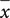 = 481 ms *vs.* 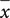 = 453 ms for *pre-* and *post-training*, respectively).

##### Executive functions

*Mazes:* total score in both Group A and Group B was significantly lower (*Z* = -2.648; *p* < 0.008 and *Z* = -3.182; *p* < 0.001, respectively) *pre-training* (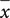 = 20.4 and 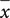 = 17.5, respectively) than *post-training* (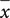 = 24.6 and 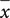 = 21.1, respectively).

*TOL^DX^:* total move score in both Group A and Group B was significantly higher (*Z* = -2.121; *p* < 0.04 and *Z* = -2.413; *p* < 0.02, respectively) *pre-training* (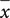 = 55.6 and 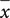 = 64.8, respectively) than *post-training* (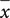 = 43.5 and 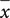 = 49.7, respectively).

#### Stability of Changes Obtained in Groups A and B

As following each training type we observed improvements in some tests, the stability of changes was assessed in Groups A and B on the basis of comparisons between *follow-up vs. post-training*. Results of these comparisons are given in **Table 4**. Due to reduced subject sample, the *follow-up* assessment was performed in 18 children (*n* = 9 in each group).

**Table 4:** Summarized results of stability of changes in Study 2 in Groups A and B for *follow-up vs. post-training* comparisons.

		Group A					Group B				
		*n*	*Post- M (SD)*	*Follow-up M (SD)*	*Z*	*p*	*n*	*Post- M (SD)*	*Follow-up M (SD)*	*Z*	*p*
TIP	ATOT	9	115 (50)	92 (63)	-1.362	ns.	9	221 (63)	193 (89)	-0.140	ns.
Language	Token Test-36	8	38.3 (23.1)	41.3 (26.8)	-0.722	ns.	9	52.6 (17.4)	52.6 (19.3)	0	ns.
	PDPseudo	6	20 (14.7)	20 (14.7)	1	ns.	7	20.8 (14.1)	23.3 (19.4)	-0.813	ns.
	PDWords	9	4.3 (6.4)	3 (5.6)	-1.841	ns.	8	7.5 (8.3)	10 (13.4)	-0.341	ns.
	SSC	8	22.5 (23.3)	15.6 (16.8)	-**2.226**	**0.03**	8	18.1 (11)	16.9 (12.8)	-0.424	ns.
	COWAT	7	8.7 (3)	11.1 (4.3)	-1.873	ns.	9	9.1 (5.3)	7.9 (4.3)	-1.199	ns.
**Other cognitive functions**									
Working	SSP	9	9.1 (2.8)	11.9 (4)	-1.436	ns.	7	12 (6.3)	10.6 (3.2)	-0.170	ns.
memory	Digit Span	5	3 (1)	3.2 (1.1)	-1.000	ns.	3	2 (1)	2.7 (1.2)	c	
	VWM	8	7.1 (1.7)	7.9 (1.7)	-1.511	ns.	6	6.7 (2.2)	7.3 (1.6)	-0.649	ns.
Attention	Alertness	7	370 (53)	400 (85)	-1.352	ns.	8	428 (121)	419 (154)	-0.980	ns.
Executive	Mazes	8	27.5 (6.5)	26.4 (5.2)	-0.853	ns.	7	22.6 (4.2)	22.3 (3.9)	-0.315	ns.
functions	TOL^DX^	8	37.8 (16.1)	33.4 (19.7)	-0.840	ns.	8	47.9 (16.8)	43.9 (15.4)	-0.763	ns.

*The significant differences were bolded. As some children in follow-up did not perform the full set of tasks, the number of tested participants is given at each tasks. c, statistical analysis was not performed because of insufficient number of subjects. Values of ATOT are provided by ms; Token Test-36, PDPseudo, PDWords, SSC, SSP by % of errors; COWAT by the number of words; Digit Span, VWM, Mazes by total score and TOL^DX^ by total move score.*

To sum up, the obtained relationships in Study 2 were relatively stable for 6 weeks after training completion. Non-significant differences between *follow-up vs. post-training* point to stable training-related changes. They were evidenced in Group A for ATOT, some language tests (Token Test-36, PDPseudo and PDWords), and other cognitive tests (SSP, Digit Span, VWM, Alertness, Mazes and TOL^DX)^). Moreover the percentage of errors in SSC in *follow-up* was significantly lower than *post-training*, indicating continued improvement. Stability of performance was also observed in Group B for COWAT, Mazes and TOL^DX^.

#### Summary of Results

The application of the temporal training (Group A) in children with SLI ameliorated significantly TIP which was reflected in lower ATOT values, as well as language skills observed in PDPseudo, PDWords, SSC and Token Test-36. Moreover, remediated working memory was evidenced in SSP, VWM and Digit Span, as well as attentional resources measured with Alertness. Finally, executive functions were also improved which was evidenced in Mazes and TOL^DX^. Following the temporal training any significant improvement was lacking in COWAT.

## Discussion

Two studies reported here measured the effects of temporal and non-temporal trainings on TIP, language skills and other cognitive functions in two language-disordered groups, i.e., in adult individuals suffering from post-stroke aphasia (Study 1) and in children with SLI (Study 2). Suggestive evidence from these studies indicated a clear dissociation between the beneficial effects of these two major intervention types, i.e., the temporal and non-temporal training. Whereas the former resulted in remediating TIP, language and other cognitive functions, the latter improved only some selected aspects of functions measured in these two studies.

### Increased Efficiency for Rapid Auditory Processing After Temporal Training

Despite a similar initial level of timing performance reflected in non-significant entrance differences in ATOT between Groups A and B in Studies 1 and 2 (see **Tables 1** and **3**), the application of two different training types (temporal or non-temporal) brought about different effects on TIP. The application of temporal training in Groups A (Studies 1 and 2) resulted in significantly lowered thresholds for the order detection *post-* than *pre-training*, corresponding with improved sequencing ability. Such difference proved non-significant in both Groups B (Studies 1 and 2), as reflected in within-group comparisons for *post vs. pre-training* performance (**Figures 3** and **4**). Divergent effects of these two major intervention types may be caused by providing various stimulation based either on rapid auditory processing (temporal training), or on a lack of such stimulation (non-temporal training).

This relationship seems independent of the content of the applied temporal intervention. Using various procedures, durations and protocols in temporal trainings in Studies 1 and 2, we found some important similarities in beneficial effects following their application. In case of aphasic patients (Study 1), we applied our prototyping, rather simple intervention program, focused on sequencing in auditory perception of event order using paired stimuli only. But in children suffering from SLI (Study 2), the more complex stimulation was applied, using the extended exercises and more attractive display. Beside ordering paired stimuli, it comprised more complex sequences of various length and presentation parameters adjusted adaptively. Moreover, an extra paradigm was implemented focusing on duration judgment in millisecond range. The novel value of these two studies is that two various contents of intervention approaches resulted in improved TIP, moreover, evidenced the transfer of improvement from the time domain into the untrained language and other cognitive domains. Although Study 1 confirms the benefits of the core idea of such therapy, the *Dr. Neuronowski*^®^ program applied in Study 2 seems more drawing a participant into the exercise, hence more attractive for the future users. It seems an optimal method of remediating language and cognitive deficits (detailed discussion below). It may be concluded that the basic content of the intervention is not a matter, but it may be important from a perspective of future users or educators.

Considering the beneficial effects it seems important to indicate a candidate mechanism that may, in concert with others, underlie ameliorated temporal acuity after the intervention. Although the direct evidence cannot be defined yet, one may hypothesize that the millisecond TIP mechanisms, responsible for sequencing abilities may operate on a very fundamental level, regardless the content of the sensory stimulation. To explain one of the possible neural sources of sequencing abilities, one may refer to neuronal spontaneous gamma band oscillations of a periodicity of 40 Hz observed in electrophysiological studies. One period of such oscillatory activity has around 25 ms duration and corresponds in duration with the time range crucial for the perception of temporal order (van Rullen and Koch, 2003). Referring to the idea proposed by Pöppel (2009), the relation ‘*before–after’* in incoming rapid events can be detected if two of them occur at least within two successive oscillatory periods. It reflects a situation when the gap between successive events falling in rapid succession is longer than one oscillatory period. The shorter gap creates problems in the proper detection ‘*before–after’* relation.

There is a strong experimental support that these neuronal oscillations play an important role in human cognition (Poeppel, 2003; van Rullen and Koch, 2003). To conclude, ameliorated TIP evidenced in lowered ATOT and improved sequencing abilities (**Figures 3** and **4**) following the temporal training may create a neural basis for remedial gains.

### Divergent Effects of Temporal and Non-temporal Training on Language Skills

The results presented here support the thesis on the close relationship between the millisecond timing and language, which was previously reported in the literature (see Introduction). The deficient millisecond timing, reflected in poorer temporal acuity evidenced in language-disordered population, may overlap with problems in speech perception/expression which is segmented temporally in the time window corresponding in duration with ATOT values.

The results obtained after temporal training in aphasic patients or after *Dr. Neuronowski*^®^ application in SLI children revealed a similar pattern of effects on sequencing abilities and language functions. Independently of the group tested (children, adults), following the temporal training we observed significant improvement of language skills which was not evidenced after the non-temporal intervention (with the exception of COWAT in Study 2). It may support a notion on a fundamental role of temporal acuity in our verbal communication. The novel important finding for clinical practice is the emphasis of the receptive language improvement in two distinct clinical groups following the intervention in millisecond range. It provides an excellent, innovatory and effective tool for the neurorehabilitation of patients suffering from receptive language problems of different etiology.

On the other hand, the novel important finding is that improved temporal acuity may result in enhanced temporal dynamic of information processing, thus, more concert processing with the typical temporal dynamic of the human speech (see Introduction). The optimization within the temporal template may create a neural basis for improved speech perception/expression which is characterized by a typical temporal segmentation. Another possibility would be a contribution of other cognitive functions, like working memory, attention or executive functions which are involved in information processing, including speech processing. These functions might be ameliorated following the intervention providing an enhancement for verbal processing. We discuss these two possibilities below.

The application of the prototypic temporal training in aphasic patients and *Dr. Neuronowski*^®^ intervention in SLI children resulted mainly in amelioration of language processes on phonemic level. These data are consistent with our previous pilot studies in patients with aphasia (Szelag et al., 2014) in which the improvement in receptive language functions was observed even after the simple temporal training. Here, we confirm the significantly better performance in both groups after various temporal trainings in phoneme discrimination tasks on syllable (PHPseudo), word (PHWords) and sentence level (ComprVC, Token Test). However, no improvement was evidenced in phonemic hearing (PHNoise, PHCompr) and inflection functions (IT).

Phoneme discrimination on syllable and word level measured in Studies 1 and 2 was based on pure auditory processing, without any extra cues. As mentioned in the Introduction, phoneme discrimination uses spectral cues which comprise rapid formant transitions in millisecond time window, similar to that critical for timing on this range. An efficient information processing within this time range is crucial for auditory comprehension on the basic level of phonemes, syllables, and words which constitute the segments of verbal utterances. Thus, improvement of temporal acuity on millisecond level, in other words – speeding up the internal clock or better synchronization of neural oscillations, resulted in more accurate phoneme discrimination. The strong correlation between language and timing was evidenced in our recent study on aphasic patients (Oron et al., 2015). It may be assumed that such temporal mechanism operates on a very basic level, regardless of the kind of material being processed (verbal/non-verbal).

Another important result obtained in both tested groups was the improvement of comprehension of spoken commands of increasing length and complexity (Token Test in Study 1 and Token Test-36 in Study 2). It required not only phoneme discrimination but involved also higher linguistic functions (semantic, syntactic and/or post interpretative processes), moreover, a strong component of visual and auditory processing or working memory load. Following temporal training we observed also better performance in the other tests involving the higher linguistic functions. In patients with aphasia (Study 1) it was evidenced in sentence comprehension (ComprVC) which comprised spatial orientation in the body schema. In children with SLI it was found in syntactic comprehension using SSC. The functions measured in Token Test, Token Test-36, ComprVC and SSC required not only well preserved linguistic processing (phonemic, semantic and syntactic) but also efficient verbal working memory. As the working memory was improved after the temporal trainings in both groups, its contribution to improved performance on these tests would be also possible (see the next section for detailed discussion).

It is interesting to note that in Study 2 in Group B we observed improved verbal fluency and executive functions measured with COWAT. This task required a spontaneous word production, as well as an ability to create, plan and execute activities. The better performance of this task may be explained referring to the strong component in the non-temporal training of expressive language skills (e.g., word presentations and repetitions) which could extended children vocabulary. Moreover, the better performance on COWAT may be associated with overall improvement in Group B in executive functions, measured with Mazes and TOL^DX^. The similar expressive language improvement after the temporal training was observed by Heim et al. (2013).

In aphasic patients after the temporal training non-significant improvement was observed in phonemic hearing using modified conditions, i.e., a background of noise (PHNoise) and compressed speech (PHComp). Such lack of improvement may be due to the specific test procedure based on pictures displaying the auditory presented sentences. Such extra visual cues might be helpful in phoneme identification. Similarly, in aphasic patients no effect was observed in auditory comprehension of grammar structures, using sentences with internal modifications in which one or more grammatical categories with declension and suffixes were presented (IT). In Polish language these grammar structures constitute the most difficult elements of language which may generate problems even in healthy language users. The constant level of performance with relatively high percentage of errors before and after the training (**Figure 3**) may reflect the task difficulty and the severity of impairment.

To sum up, temporal trainings improve language functions in two distinct clinical groups, suggesting the coexistence of the common neural platform which control information processing. In case of non-temporal training these beneficial effects were generally not found.

### Divergent Effects of Temporal and Non-temporal Training on the Other Cognitive Functions

The positive temporal training effects widespread for the other cognitive functions, i.e., working memory, attention and executive functions. In Study 1 we observed a clear remediation of working memory capacity. After the temporal training patients memorized significantly more items (SSP) and committed significantly fewer errors in SWM. Such improvements may result from improved TIP and higher temporal acuity (see above) which created a modified frame for working memory, as well as other cognitive functions (Szelag et al., 2011b; Bao et al., 2013). Some authors (Ulbrich et al., 2009) emphasized the relations between millisecond timing and working memory. Although in aphasic patients during the temporal training working memory was not trained directly, the changes within a temporal template underlying our mental activity may modify the working memory resources. As a consequence, the remediated working memory may facilitate the performance on other tasks, including some language tasks applied in Study 1 (Token Test, ComprVC) or executive function tasks in Study 2 (Mazes, TOL^DX^) in which the load of working memory is high. A number of existing evidence confirmed the contribution of working memory to cognition, including speech processes (see Baddeley, 2003 for a review). Moreover, to succeed in the training based on temporal ordering, the trained temporal skills had to be accompanied by the efficient working memory load.

Improved working memory was also observed after the application of *Dr. Neuronowski*^®^ program in children with SLI (Study 2) which was evidenced in lower errors in SSP, increased total score in VMM and Digit Span (**Figure 4**). As *Dr. Neuronowski*^®^ is much more extended tool compared to the intervention applied in aphasic patients, it provided more massive stimulation addressing many cognitive functions directly, including working memory, attention and executive functions.

According to Lezak (1995), attention functions differ from other cognitive functions and they could be treated as mental activity variables which are highly engaged in many other cognitive functions. In children with SLI, decreased RTs in Alertness were observed after the temporal training. In this terms, shorter RT corresponding with better performance, may reflect the increased general processing speed (van Zomeren and Bouwer, 1994). Some authors (Stevens et al., 2008) suggested that improvement in receptive language in children with SLI after administration of FFW may be caused by the enhancement in sustained attention. It should be stressed that the correlation between some aspect of attention and TIP was observed also in our previous studies (Szymaszek et al., 2009; Oron et al., 2015).

Evidence suggest that children with SLI display difficulties in tasks engaging executive functions (e.g., Roello et al., 2015). Alarcón-Rubio et al. (2014) showed that receptive vocabulary skills and self-directed speech usage are associated with executive functions in typically developing children aged from 4 to 7 years. Such relationship may indicate the similar temporal frame provided by the temporal mechanisms on which various cognitive functions are embedded. Such hypothesis finds it support in taxonomy of neuropsychological functions provided by Pöppel (1994) which assumes that TIP provides a logistic basis for many cognitive activities.

In literature data the relationship ‘*TIP-executive functions’* is a neglected topic. It inspired us to include executive functions into the diagnostic set in Study 2. Interestingly, the improvement of executive functions was evidenced in Mazes and TOL^DX^ following the temporal training (Group A) and non-temporal one (Group B). In our opinion, such improvement in Groups A and B may result from different reasons. In Group A it may result from the improvement of the temporal dynamic of the neural network (see above for explanations), direct training of the executive functions (*Module 4* and *8*), or from interaction between these two factors. In contrast, in Group B in Study 2 remediating executive functions might result from playing computer games which were included into some parts of our non-temporal intervention. Its beneficial effects with respect to the mental activity were previously reported by Bavelier et al. (2011).

To conclude, the improved cognition may result from the interrelation between the improved temporal frame and cognitive load contained in the applied intervention in parallel to the TIP exercises. Beneficial effects of temporal and non-temporal training for the mental activity supports the contribution of other cognitive functions to speech therapy.

### Implications for Actual Practice and Future Research

The current report has important implications for clinical practice and future experimental studies. The novel value of two studies presented here is the indication for the first time that two distinct language disorders of various etiologies, i.e., post-stroke aphasia in adults and SLI in children which are characterized by various profiles of language impairment may be remediated by a similar intervention program based on non-verbal training in TIP. Specifically, post-stroke receptive aphasia investigated in Study 1 is characterized by relatively fluent verbal output but disordered auditory comprehension. In contrast, SLI investigated in Study 2 is characterized by developmental language production and/or comprehension deficits (usually mixed) that cannot be explained by general cognitive impairment, concomitant impairments or a general lack of exposure to language. Whereas the etiology of aphasia is usually well defined and the lesioned area may be evidenced in neuroimaging examination (see **Figure 1**), the etiology of SLI is difficult to define and remains predominantly unknown.

The exciting phenomenon of human language is that, despite the totally different pattern of language impairment in case of these two disorders, there are some important similarities in neuronal mechanisms underlying disordered language. These mechanisms are rooted in temporal acuity on millisecond range and can be studied using both verbal (temporal dynamic of the spontaneous speech) or non-verbal information processing (indexed by ATOT). The important finding is that these distinct language impairments at least in some cases are sensitive to the specific training focused on TIP. Such finding can help to design and elaborate future remediation programs supporting the classic speech therapy.

A final question is who more could benefit from such therapy program. In our previous studies (Szelag and Skolimowska, 2012) we indicated that the temporal frame does not underlie selectively speech processing, but also some other cognitive functions, like working memory, attention or executive control which can be characterized also by the specific temporal dynamics. Moreover, these cognitive functions could be also remediated following the specific training (**Figures 3** and **4**, see the Results). We conclude, therefore, that the future horizons for the application of the temporal intervention may be expanded by applications in enhancement of the broad aspects of cognitive functioning. Such view point may be supported by the results of our previous study in which we indicated that the application of FFW training in normal healthy elderly beyond 65 years of life resulted in improved attention and short-term memory (Szelag and Skolimowska, 2012).

To sum up, amelioration of disordered timing can be used as an universal tool in future clinical practice not only in language-disordered population, but also in people with various cognitive dysfunctions.

## Conflict of Interest Statement

The authors declare that the research was conducted in the absence of any commercial or financial relationships that could be construed as a potential conflict of interest.
