# Corrigendum: The Application of Timing in Therapy of Children and Adults with Language Disorders

**DOI:** 10.3389/fpsyg.2016.00449

**Published:** 2016-03-30

**Authors:** Elzbieta Szelag, Anna Dacewicz, Aneta Szymaszek, Tomasz Wolak, Andrzej Senderski, Izabela Domitrz, Anna Oron

**Affiliations:** ^1^Laboratory of Neuropsychology, Nencki Institute of Experimental BiologyWarsaw, Poland; ^2^University of Social Sciences and HumanitiesWarsaw, Poland; ^3^Institute of Physiology and Pathology of HearingKajetany, Poland; ^4^Children's Memorial Health InstituteWarsaw, Poland; ^5^Department of Neurology, Warsaw Medical UniversityWarsaw, Poland

**Keywords:** temporal information processing, language, cognitive functions, aphasia, specific language disorder

Conflict of Interest Statement

ES and AS are the creators of the software package Dr. Neuronowski®, realized as part of a project at the Nencki Institute with funding from the National Centre for Research and Development in Poland. The rights to the software lie with the Nencki Institute that has an agreement with Harpo Ltd., the company commercializing this software. ES and AS are not the owners of this technology nor do they have a direct financial arrangement with Harpo Ltd.

The authors state that the correction does not affect the scientific validity of the results.

## Author contributions

ES: experimental design, data acqusition and analysis, manuscript writing. AD: data acqusition and analysis, manuscript writing. AS: data acqusition and analysis, manuscript writing. TW: guidance on MRI data analysis. AS: subject recruitment. ID: subject recruitment. AO: data acqusition and analysis, manuscript writing.

### Conflict of interest statement

The other authors declare that the research was conducted in the absence of any commercial or financial relationships that could be construed as a potential conflict of interest.

